# Dietary sodium to potassium ratio is an independent predictor of cardiovascular events: a longitudinal follow-up study

**DOI:** 10.1186/s12889-023-15618-7

**Published:** 2023-04-18

**Authors:** Zahra Mosallanezhad, Mohammad Jalali, Zahra Bahadoran, Parvin Mirmiran, Fereidoun Azizi

**Affiliations:** 1 Nutrition and Endocrine Research Center, Research Institute for Endocrine Sciences, Shahid Beheshti University of Medical Sciences, No. 24, Shahid-Erabi St., Yeman St, P.O.Box: 19395-4763, Velenjak, Tehran, Iran; 2 Department of Clinical Nutrition and Dietetics, Faculty of Nutrition and Food Technology, National Nutrition and Food Technology Research Institute, Shahid Beheshti University of Medical Sciences, Tehran, Iran; 3 Endocrine Research Center, Research Institute for Endocrine Sciences, Shahid Beheshti University of Medical Sciences, Tehran, Iran

**Keywords:** Sodium, Potassium, Sodium to potassium ratio, Cardiovascular disease

## Abstract

**Background:**

The current prospective cohort study aimed to explore the potential associations between dietary sodium (Na), potassium (K), and sodium-to-potassium (Na-to-K) ratio with an incidence risk of cardiovascular disease (CVD) among Iranian adults.

**Methods:**

The participants of the Tehran Lipid and Glucose Study (men and women aged 30–84 years, n = 2050), free of CVD at baseline (2006–2008) were included. Dietary intakes were assessed using a validated food frequency questionnaire (FFQ), and incident CVD (i.e., coronary heart disease, stroke, and CVD mortality) were documented up to March 2018. Cox proportional hazard models were used to estimate hazard ratios (HRs) and 95% confidence interval (CI) regarding the association between dietary Na, K, and Na-to-K ratio with CVD events.

**Results:**

During a median follow-up of 10.6 years, 10.14% of participants experienced CVD outcomes. A 41% increased risk of CVD in relation to each increase in 1000 mg/d of Na intake. In the fully-adjusted model, higher Na intake (> 4143 versus < 3049 mg/d) was significantly related to the increased risk of CVD (HR = 1.99, 95% CI = 1.06–3.74). Independent of the well-known risk factors, a 56% reduced risk of CVD was observed in the participants with a higher dietary K intake (HR = 0.44, 95% CI = 0.20–0.94). A Higher Na-to-K ratio was associated with an increased risk of CVD (HR = 1.99, 95% CI = 1.13–3.52).

**Conclusion:**

Our study showed that the Na-to-K ratio might independently predict future risk of CVD events in adults.

**Supplementary Information:**

The online version contains supplementary material available at 10.1186/s12889-023-15618-7.

## Introduction

Cardiovascular disease (CVD) is a major cause of death globally [1]. Metabolic risk factors including hypertension (HTN), diabetes mellitus and dyslipidemia play an important role in the etiology of CVD, among which, HTN is considered as the major and preventable risk factor for CVD mortality and disability [1, 2]. The number of diet-attributable CVD deaths and disability-adjusted life years (DALYs) were 6.9 million and 153.2 million, in 2019, marking a 43.8 and 34.3% rise from 1990, respectively [3]. Dietary sodium (Na) and potassium (K) are the most important dietary components that are associated with HTN, CVD and other health outcomes [4]. High consumption of Na along with low dietary K, particularly in Western diets high in refined grain, added sugar, sweetened drinks and processed food, and low in fruit and vegetable, have a synergistic effect on development of HTN [5]. World Health Organization (WHO) recommends that consumption of less than 2000 mg of Na (2-gr Na ~ 5-gr salt) and at least 3510 mg of K per day to reduce HTN, incidence of CVD and coronary heart disease (CHD) [6–8]. In addition, the dietary guidelines for Americans (DGA) recommends that Na intake should be limited to less than 2.3 g/d in general population and 1.5 g/d in HTN and diabetes [7]. The global estimation of mean intake of salt is around 2- to 3-fold greater than the recommendations and almost 95% of people consume between 6–12 g/d [9]. Despite available evidence regarding harms of “excessive” sodium (Na) intake, the term is still remained undefined, and new evidence is going to critically change the conventional belief [10–14]. The cause-and-effect relationship between Na intake and risk of CVD still remains inconsistent; strong positive associations [15], U-shaped [16] and null association [10, 11] have been reported among different populations. There is an interaction between Na and K intakes in relation to the risk of CVD [17], and Na-to-K ratio may be a more accurate predictor of CVD risks than dietary Na or K per se [14]. Previous studies showed that dietary Na-to-K ratio that is strongly correlated with urinary Na and K concentrations, predicted the risk of developing CVD, HTN, ischemic strokes, and chronic kidney disease [2, 5, 7, 18, 19].

The present cohort study aimed to investigate the potential cumulative effects of Na, K and their ratio, obtained by repeated dietary assessments using food frequency questionnaires (FFQ), with CVD over 10.6 years follow-up.

## Methods

### Study design and population

The present cohort study was conducted in the framework of the Tehran Lipid and Glucose Study (TLGS) [20]. In brief, TLGS is an ongoing population-based prospective study started from 1999 to 15,005 individuals aged ≥ 3 years old, resident in the Tehran, capital city of Iran. For the current analysis, CVD-free adult (≥ 30 years old) participants of the third phase of TLGS (2006–2008), with completed data on demographics, clinical, biochemical and dietary assessments were included (n = 2050), and followed-up to March 2018.

This study was done according to the Helsinki declaration of ethics. Informed written consent was obtained from each participant, and the ethical committee of Shahid Beheshti University of Medical Sciences., Tehran, Iran, approved the study protocol.

### Assessment of covariates

A pre-defined questionnaire was used to collect the participants’ information including sex, age, smoking habits, medications, and menopause status, personal and family history of diseases. A validated Modifiable Activity Questionnaire (MAQ) for Iranian was used to assess the physical activity status [21]. Weight was measured to the nearest 100 g using digital scales, while the subjects were minimally clothed, without shoes. Height was measured to the nearest 0.5 cm, in a standing position without shoes, using a tape meter. Body mass index (BMI) was calculated as weight (kg) divided by square of the height (m^2^). Waist circumference was measured to the nearest 0.1 cm, midway between the lower border of the ribs and the iliac crest at the widest portion, over light clothing, using a soft measuring tape, without any pressure to the body. Systolic blood pressure (SBP) and diastolic blood pressure (DBP) were determined after 15 min of rest in a seated position on the right arm using a calibrated mercury sphygmomanometer (Riester, Germany) [22]. Measurement was repeated twice with the interval of 30 s, and mean of them was considered as the participants’ blood pressure.

Details of biochemical measurements in the TLGS samples have been described elsewhere [23]. In brief, biochemical measurements [i.e., fasting serum glucose (FSG), triglyceride (TG), and high-density lipoprotein cholesterol (HDL-C] levels were all done after a 12-to 14-h overnight fast, at baseline and all subsequent examinations (with a 3-y follow-up intervals). The standard oral glucose tolerance test (OGTT) was performed for all adults (age ≥ 21 y) who were not on glucose-lowering medications. Serum glucose concentration was measured with an enzymatic colorimetric method using glucose oxidase (Pars Azmoon, Tehran, Iran). Serum Cr concentrations were determined by the Jaffe kinetic alkaline picrate method. Intra- and inter-assay coefficients of variation (CV) were less than 5.0% for all measurements.

### Dietary assessment

Dietary intakes over the last year were collected using a validated 168-items food frequency questionnaire (FFQ) [24]. Participants were asked to report the frequency (e.g. daily, weekly, or monthly); portion size (e.g. cup, spoon, and ounce) of each consumed food were then converted to grams. Mean intake of Na and K during the last year was provided by FFQ. As the Iranian Food Composition Table (FCT) is incomplete and has limited data on the content of raw foods and beverages’ nutrient, the US Department of Agriculture (USDA) FCT was used. For national foods not listed in the USDA FCT, the Iranian FCT was used [25].

### Definition of outcomes and terms

The data collection and definition of CVD outcome has been previously described by the TLGS research group [26, 27]. The international statistical classification of diseases and related health problems, 10th Revision CRITERIA, and the American heart association classification for cardiovascular events were used for definition of the outcomes. As described by Hadaegh et al. [26, 27], CVD was defined as any coronary heart disease (CHD)-related event, stroke, or CVD death [definite fatal myocardial infarction (MI), definite fatal CHD, and definite fatal stroke) (26, 28). The CHD-related events were considered as cases of definite MI (according to diagnostic ECG and biomarkers), probable MI (according to positive ECG findings, cardiac symptoms or signs, and missing biomarkers, or positive ECG findings and equivocal biomarkers), unstable angina pectoris (new cardiac symptoms or changing symptom patterns and positive ECG findings with normal biomarkers), angiographic-confirmed CHD, and CHD death. Death from CHD or stroke was confirmed by reviewing the death certificate or medical records. Stroke was considered as a new neurological deficit that lasted at least 24 h [26, 27].

Type 2 diabetes mellitus (T2DM) was defined as fasting serum glucose ≥ 126 mg/dL, 2-h serum glucose ≥ 200 mg/ dL, or using anti-diabetic medications [29]. Elevated systolic blood pressure (SBP) ≥ 140 mmHg, elevated diastolic blood pressure (DBP) ≥ 90 mmHg, or using antihypertensive drugs were considered as HTN [30].

The CKD Epidemiology Collaboration (EPI) equation was used to calculate estimated glomerular filtration rate (eGFR). As a single equation CKD-EPI has been expressed as follows: eGFR = 141×min (S_cr_/κ, 1)^α^ × max (S_cr_/κ, 1)^−1.209^ × 0.993^age^ × 1.018 [if female] × 1.159 [if black]. In this equation, S_cr_ is serum creatinine in mg/dL; κ is 0.7 and 0.9 for men and women, respectively, α is -0.329 and − 0.411 for men and women; min indicates the minimum of S_cr_/κ or 1, and max indicates a maximum of S_cr_/κ or 1 [31].

### Statistical analysis

Baseline characteristics of the study participants were described by mean ± standard deviation (SD) values, frequency (%), or median (inter-quartile range, IQR) across CVD status. Continuous variables with normal distribution were compared between the groups using independent t-test whereas dichotomous variables were compared using chi-square test. To compare variables with non-normal distributions, the Mann-Whitney U test was used.

The hazard ratio (HR) and 95% CI for the incidence of CVD were reported as continuous (per 1000 mg/d increase in Na and K) and categorized (tertiles). Time to event was defined as time from baseline (2006–2008) to end of follow-up (censored cases) or time to having an event, whichever occurred first. The proportional hazards assumption was tested. We censored participants at the time of death due to non-CVD causes, at the time of leaving the district, or study follow-up end time of March 2018.

Two Cox models were planned; model 1 was controlled for CVD risk score, which was included a sex-specific general CVD algorithms incorporating age, total cholesterol, HDL-C, SBP, treatment for HTN, smoking and T2DM [32]. The accuracy and clinical importance of this score for our population was also reported in detail [33]. Model 2 was additionally adjusted for physical activity, energy intake, dietary fat and fibers. Model 3 was additionally adjusted for eGFR and menopause status (yes/no).

The receiver operator characteristic (ROC) curve analysis was used with an estimation of the variable sensitivity and specificity to determine the cut-off point of dietary Na-to-K ratio for risk of developing CVD. The cut-off point was assessed by pre-defined value of sensitivity (i.e., 0.7). To obtain the optimal cut-off dietary Na-to-K ratio for risk of developing CVD, Youden index (the maximum value of sensitivity + specificity–1) was used, the index is preferable for the finding of the optimal cut-off point because it is clinically translated to maximizing correct classification and minimizing misclassification rates [34].

We used Statistical Package for Social Science (SPSS) (version 22; IBM Corp., Armonk, NY, USA) for all analyses. *P* values < 0.05 were considered as statistical significance.

## Results

A total of 2050 participants (46% male) with the mean (SD) age of 46.28 (11.34) were included to the analysis. Median (interquartile range) of follow-up duration was 10.6 (9.9–11.1) years, the incidence rate of CVD events during that time was 10.14%. In comparison with non-CVDs, participants who developed CVD outcomes were more likely to be older, male and had higher WC, SBP, DBP, TG to HDL-C ratio, serum total cholesterol and fasting serum glucose levels (*P* for all < 0.001). No significant differences in other sociodemographic characteristics, including BMI, current smoking, physical activity and Na-to-K ratio were observed **(**Table 1**)**.

**Table 1:** Baseline characteristics of the study participants (n = 2050)

Baseline Characteristics	Total (n = 2050)	CVD^+^ (n = 208)	CVD^−^ (n = 1842)	*P*
Age (year)	46.28 ± 11.34	57.24 ± 10.15	45.04 ± 10.79	0.001
Male (%)	46.0	65.9	43.8	0.001
BMI	28.08 ± 4.62	28.62 ± 4.76	28.02 ± 4.61	0.08
WC (cm)	92.9 ± 11.98	98.2 ± 11.24	92.3 ± 11.91	0.001
HTN (%)	16.9	38.6	14.5	0.001
SBP	114 ± 17.72	127 ± 20.82	113 ± 16.71	0.001
DBP	75.2 ± 10.81	79.9 ± 12.84	74.7 ± 10.43	0.001
Serum cholesterol	195 ± 37.90	208 ± 41.54	193 ± 37.19	0.001
Diabetes (%)	9.2	20.2	7.9	0.001
FSG (mg/dL)	94.97 ± 25.95	106 ± 37.61	93.6 ± 23.94	0.001
Current Smoking (%)	19.6	24.5	19.0	0.06
Physical activity* (MET-hours/weeks)	15.87 (4.24–39.69)	13.89 (3.96–34.96)	16.12 (4.27–40.12)	0.53
TG to HDL ratio	4.23 ± 3.30	4.92 ± 3.01	4.15 ± 3.33	0.001
Na-to-K ratio	1.18 ± 0.67	1.20 ± 0.65	1.18 ± 0.67	0.70

Data are mean ± SD unless stated otherwise (independent t-test and chi-square test were used for continuous and dichotomous variables, respectively. Mann-Whitney U test was applied to compare variables without normal distributions)

* Data is median (IQR)

* CVD (cardiovascular disease); BMI (body mass index); WC (waist circumference); HTN (hypertension); SBP (systolic blood pressure); DBP (diastolic blood pressure); FSG (fasting serum glucose); TG (triglyceride); HDL (high density lipoprotein lipase); Na (sodium); K (potassium)

The mean dietary Na and K intake were 4200 and 3790 mg/d, respectively. The baseline dietary intakes of participants across tertiles of the Na intakes are presented in Table 2. Dietary intakes of energy, protein, carbohydrates, fats, cholesterols, saturated fats, fibers, fruits and vegetables, potassium, refined grains, whole grains, dairy products, salty snacks, fast foods, red meats and poultry were higher in the third versus first tertiles (*P* for all < 0.001).

**Table 2:** Dietary intakes of participants across tertiles Sodium intake: Tehran Lipid and Glucose Study

Dietary intakes	Total (n = 2050)	Tertiles of Sodium intake			*P*
		T1 (n = 683)	T2 (n = 684)	T3 (n = 683)	
Total energy intake (kcal/day)	2273 ± 685	1883.41 ± 508	2305 ± 609	2630 ± 705	0.001
Total Protein intake (g/day)	77.3 ± 24.35	64.6 ± 18.38	78.9 ± 22.43	88.4 ± 25.56	0.001
Total Carbohydrate intake (g/day)	328 ± 104	274 ± 80.8	331 ± 94.2	379 ± 108	0.001
Total fat intake (g/day)	77.4 ± 26.57	63.7 ± 20.53	78.7 ± 24.59	89.7 ± 27.49	0.001
Total cholesterol intake (mg/day)	210 ± 86.0	175 ± 73.2	219.37 ± 82.15	237 ± 90.0	0.001
Saturated fat intake (g/day)	25.52 ± 9.41	21.23 ± 7.76	26.09 ± 8.94	29.22 ± 9.66	0.001
Total Fiber intake (g/day)	38.05 ± 16.79	30.17 ± 12.21	38.44 ± 16.03	45.54 ± 17.93	0.001
Fruits & vegetables (g/day)	701 ± 333	630 ± 310	696.46 ± 321.70	777 ± 349	0.001
Total Sodium intake (g/day)	4.20 ± 2.08	2.46 ± 0.58	3.72 ± 1.25	6.43 ± 1.68	0.001
Total Potassium intake (g/day)	3.79 ± 1.29	3.27 ± 1.09	3.83 ± 1.21	4.28 ± 1.34	0.02
Refined grains (g/day)	312 ± 138	273 ± 125	311.70 ± 136.24	352 ± 142	0.001
Whole grains (g/day)	86.5 ± 65.7	68.7 ± 53.5	93.33 ± 67.24	97.5 ± 71.5	0.001
Dairy (g/day)	449 ± 223	403 ± 209	465.70 ± 225.21	479 ± 229	0.05
Salty snacks (g/day)	7.75 ± 7.80	5.16 ± 6.03	8.38 ± 7.81	9.70 ± 8.63	0.001
Fast foods (g/day)	13.64 ± 10.56	10.66 ± 9.13	14.54 ± 10.55	15.71 ± 11.24	0.001
Red meats (g/day)	16.48 ± 11.96	14.09 ± 10.75	16.58 ± 11.90	18.78 ± 12.72	0.001
Poultry (g/day)	35.32 ± 21.27	31.11 ± 19.45	36.72 ± 21.47	38.14 ± 22.18	0.001

Data are mean ± SD, ANCOVA was used to adjust for energy intakes

The risk of CVD across tertile categories of Na, K, and Na-to-K ratio are shown in Table 3. Continuous variable analysis showed a 41% increased incidence of CVD associated with each 1000 mg/d increase in dietary Na (HR = 1.41, 95% CI = 1.11–1.79) in the full model. After adjustment of CVD risk score, higher Na intake (> 4143 versus < 3049 mg/d) was significantly related to the increased risk of CVD (HR = 1.41, 05% CI = 1.03–1.94). Further adjustment for physical activity, energy intake, dietary fiber and fats, eGFR and menopause status, the association was potentiated (HR = 1.99, 95% CI = 1.06–3.74). In addition, fully-adjusted model showed a lower risk of CVD for 56% in participants with the highest K intake in compared to lowest tertile (HR = 0.44, 95% CI = 0.20–0.94) **(**Table 3**)**. Continuous variable analysis showed a 29% decreased incidence of CVD associated with each 1000 mg/d increase in dietary K (HR = 0.71, 95% CI = 0.52–0.97) in the full model.

**Table 3:** The risk of CVD events across tertiles categories of dietary sodium, potassium and sodium-to-potassium ratio

	Tertiles of Sodium and potassium intake							Per 1000 mg intake	
	T1 (n = 683)			T2 (n = 684)		T3 (n = 683)			
**Sodium**									
*Crude*		1.00	0.70 (0.49-1.01)		1.23 (0.90-1.69)		1.18 (1.03-1.35)^*^		
*Model1*		1.00	0.74 (0.52-1.06)		1.41 (1.03-1.94)^*^		1.23 (1.08-1.41)^**^		
*Model2*		1.00	0.86 (0.58-1.27)		1.83 (1.25-2.68)^**^		1.42 (1.22-1.66)^**^		
*Model3*		1.00	0.92 (0.46-1.85)		1.99 (1.06-3.74)^**^		1.41 (1.11-1.79) ^**^		
**Potassium**									
*Crude*		1.00	0.70 (0.50-0.98)^*^		0.78 (0.56-1.08)		0.93 (0.81-1.06)		
*Model1*		1.00	0.70 (0.50-0.98)^*^		0.75 (0.54-1.04)		0.91 (0.80-1.04)		
*Model2*		1.00	0.68 (0.47-0.98)^*^		0.64 (0.42-0.97)^*^		0.86 (0.72-1.02)		
*Model3*		1.00	0.65 (0.35-1.19)		0.44 (0.20-0.94)^*^		0.71 (0.52-0.97) ^*^		
**Sodium/Potassium**									
*Crude*		1.00	0.96 (0.67-1.37)		1.51 (1.08-2.09)^*^				
*Model1*		1.00	1.08 (0.75-1.55)		1.73 (1.24-2.40)^**^				
*Model2*		1.00	1.25 (0.85-1.84)		2.03 (1.42-2.90)^**^				
*Model3*		1.00	0.87 (0.44-1.71)		1.99 (1.13-3.52) ^*^				

Tertile 1 was considered as reference. Cox regression models were used. Model 1: Adjusted for CVD-risk score; Model 2: Additionally adjusted for physical activity, energy intakes (kcal/d), dietary intakes of total fats (g/d), and fiber (g/d); Model 3: additionally adjusted for eGFR and menopause status (yes/no).

median) Sodium intake (mg/d) range and median across tertiles: T1: <3049 (2573); T2: 3049-4143 (3566); T3: >4143 (4977)

Range ( Potassium intake (mg/d) range and median across tertiles: T1: <3489 (3335); T2: 3489-4660 (4198); T3: >4660 (4684)

Na-to-K ratio range and median across tertiles: T1: <0.79 (0.78); T2: 0.79-1.02 (0.87); T3: >1.02 (1.15)

* *p* <0.05. ** Statistical significance at *p* <0.01

After adjustment of CVD risk factors, an increased risk of CVD events was observed in the participants with highest compared to lowest dietary Na-to-K ratio (HR = 1.73, 95% CI = 1.24–2.40). Additional adjustment for physical activity, energy intake, dietary fiber and fats, eGFR and menopause status increased the CVD incident risk in relation to higher dietary Na-to-K ratio (HR = 1.99, 95% CI = 1.13–3.52) **(**Table 3**)**.

The critical cut-off point of Na-to-K ratio for predicting CVD events was 0.80 (at a fixed sensitivity of 70%, AUC = 0.56, 95% CI 0.54–0.58, *P* value = 0.001) (Supplementary Fig. 1). In the presence of traditional CVD risk factors, dietary Na-to-K ratio higher than the cut-off value (≥ 0.80) was related to increased risk of CVD by % (HR = 1.63, 95% CI = 1.17–2.27). The calculated Youden index addressed a cut-off point of 1.26 for Na-to-K ratio (sensitivity = 29.3 and specificity = 84.3); participants with a ratio higher than the 1.26 were estimated to have an elevated risk of CVD by2.62-fold (95% CI = 1.91–3.59).

## Discussion

The present study investigated the prospective association between dietary intakes of Na, K, and Na-to-K ratio with the incident risk of CVD over 10.6 years of follow-up among an Iranian population. Our results showed that an increased risk of CVD events in relation to high intake of Na and Na-to-K ratio, and lower risks in people with higher intake of dietary K. The associations were independent of the well-established risk factors of CVD in our population. Moreover, our results showed that a 41% increased risk of CVD in relation with each increase in 1000 mg/d of Na intake. Using a ROC curve analysis with a fixed sensitivity of 70% and calculating Youden index, a cut-off value of 0.80 and 1.26 was determined as a critical and optimal cut-off points of dietary Na-to-K ratio, respectively; higher dietary Na-to-K ratio higher than the cut-off values was associated with an elevated risk of CVD by 63 and 162%, respectively.

Our findings are in agreement with previous reports of some nation-wide cohorts. The Prospective Urban Rural Epidemiology study reported an increased risk of CVD and stroke only in communities whose daily Na intake was greater than five g/d [35]. A high-Na diet (> 6 g/d) in diabetics with poorly controlled blood glucose in Japan Diabetes Complications Study (JDCS) cohort was associated with an elevated risk of CVD by ~ 10-fold, while in good-controlled patients Na intake was not related with developing CVD [15]. Recent studies suggested that direct relation between Na intake and CVD risk independent of HTN-related mechanism [36–38]. A high-Na diet causes endothelial dysfunction by reducing nitric oxide (NO) bioavailability, leading to development of atherosclerosis and cardiovascular events [37, 39]. Excessive Na intakes decreases arterial compliance and induces arterial fibrosis by increasing transforming of growth factor (TGF)-β and overproduction of reactive oxygen species [40, 41].

Mean intake of K in our population (i.e., 3790 mg/d) meets the WHO recommendation [8]. The protective effect of dietary K against development of CVD in our population was similar to previous reports linking higher dietary and urinary K with a reduced risk of CVD [42] [43], especially in those who had a high-Na intake [44, 45]. Interestingly, the Northern Manhattan study found a positive association between potassium intake and stroke among those with < 2300 mg/day sodium intake; and an inverse association among those with ≥ 2300 mg/day sodium intake [17].

In agreement with our result, NOMAS cohort reported that higher Na-to-K ratio increased risk of developing ischemic stroke by 60% [17]. A recent meta-analysis of population-based studies reported a pooled estimated relative risk of stroke by 1.22 (95% CI = 1.04, 1.41) for a one-unit increased in dietary Na-to-K ratio (mmol/mmol) [46]. The authors also reported that a urinary Na-to-K ratio of ≤ 1 may be associated with a clinically relevant reduction in stroke risk and represents a potential target for health interventions [47]. It is highly recommended to lower Na-to-K ratio and improve cardiometabolic health following greater consumption of dietary K sources such as whole grains, low-fat dairy products, fruits and vegetables [48, 49]. K intakes also reduce blood pressure by relaxing blood vessels and excreting Na through urine [38, 50].

Mean intake of salt among different populations was reported between 0.5 and 55 g/d [51, 52]. A recent national report of estimated Iranian salt intake indicated a mean population of 9.52 g/d (95% CI = 9.48–9.56) and ~ 97.7% overconsumption of salt among the population [53]. Although it can be different among populations, about 75% of dietary Na is attributed to processed foods, 10–12% is naturally occurring in foods, and the rest of 10–15% is discretionary salt intake (salt used in home-cooking or at the table) [54, 55]. In our population, Na intakes has been estimated to be originated mainly from salty-snacks and processed foods, and urinary Na was strongly correlated with the Wester dietary pattern [18].

The prospective design of our study, relatively large sample size and long-term follow-up are considered as the strength of current study. Using CVD risk score allowed us to account for major traditional CVD confounders without adding many variables and improving our models’ instability [33]. In addition, we used a validated FFQ to estimate usual dietary intakes of the Iranian population. The present study has potential limitations. We used dietary intakes for variables of interest rather than urine collections, which more accurately reflect the status of Na-to-K ratio. Additionally, we were unable to capture table salt intake, as the important source of dietary Na. As inherent in any prospective study, some degree of misclassification might have occurred during study follow-up due to possible changes in an individual’s diet as well as changes in confounders. Finally, as the questionnaires we used were self-repots, potential measurement errors in estimated of food and nutrient intakes might accrues.

To sum up, our results provided further support for previous studies reported adverse effect of high-Na intake and favorable effect of dietary K on the cardiovascular function. We also showed that Na-to-K ratio may independently predict future risk of CVD events in adult population. A balanced Na-to-K ratio needs to be considered to prevent diet-induced secondary hypertension as a risk factor of cardiovascular disease.

## Electronic supplementary material

Below is the link to the electronic supplementary material.

Supplementary Material 1

## Data Availability

Data will be presented upon forwarding the request to the corresponding author (z.bahadoran@endocrine.ac.ir) and confirmation of the director of RIES (azizi@endocrine.ac.ir).
