# Supplementary material for: Dietary sodium to potassium ratio is an independent predictor of cardiovascular events: a longitudinal follow-up study
Source: BMC Public Health. 2023 Apr 18;23:705. doi: 10.1186/s12889-023-15618-7 (PMC10111692; doi:10.1186/s12889-023-15618-7)
Supplement: Supplementary file 1 — Supplementary Material 1 [file 12889_2023_15618_MOESM1_ESM.docx]

**Supplementary Figure 1.** Estimated cut-off point of Ox-to-Ca ratio for incident cardiovascular disease (0.80, at a fixed sensitivity of 70%, AUC =0.56, 95% CI 0.54-0.58, *P* value=0.001; 1.26, sensitivity=29.3 and specificity=84.3).
